# The Fragility of Statistical Findings in Cervical Disc Arthroplasty: a Systematic Review of Randomized Controlled Trials

**DOI:** 10.1007/s00402-024-05353-y

**Published:** 2024-05-03

**Authors:** Emmanuel C. Megafu, Michael N. Megafu, Janet T. Nguyen, Elisabeth Point Du Jour, Wesley H. Bronson, James D. Lin, Andrew C. Hecht, Robert L. Parisien

**Affiliations:** 1 Geisinger Commonwealth School of Medicine, Scranton, PA USA; 2 A.T. Still University, Kirksville College of Osteopathic Medicine, Kirksville, MO USA; 3 Department of Orthopedic Surgery, Mount Sinai Hospital, New York, NY USA

**Keywords:** Statistical fragility, Cervical disc arthroplasty, Randomized controlled trials, Fragility index, Fragility quotient

## Abstract

**Purpose:**

This study employs both the fragility index (FI) and fragility quotient (FQ) to assess the level of robustness in the cervical disc arthroplasty (CDA) literature. We hypothesize that dichotomous outcomes involving CDA would exhibit statistical vulnerability.

**Methods:**

A PubMed search was conducted to evaluate dichotomous data for randomized controlled trials (RCTs) in CDA literature from 2000 to 2023. The FI of each outcome was calculated through the reversal of a single outcome event until significance was reversed. The FQ was calculated by dividing each fragility index by the study sample size. The interquartile range (IQR) was also calculated for the FI and FQ.

**Results:**

Of the 1561 articles screened, 111 met the search criteria, with 35 RCTs evaluating CDA included for analysis. Six hundred and ninety-three outcome events with 130 significant (*P* < 0.05) outcomes and 563 nonsignificant (*P* *≥* 0.05) outcomes were identified. The overall FI and FQ for all 693 outcomes were 5 (IQR 3–7) and 0.019 (IQR 0.011–0.043). Fragility analysis of statistically significant outcomes and nonsignificant outcomes both revealed an FI of 5. All of the studies reported loss to follow-up (LTF) data where 65.7% (23) did not report or reported an LTF greater or equal to 5.

**Conclusions:**

The literature regarding CDA RCTs lacks statistical robustness and may misrepresent the conclusions with the sole use of the *P* value. By implementing the FI and FQ along with the *P* value, we believe the interpretation and contextualization of the clinical data surrounding CDA will be better understood.

## Introduction

Cervical disc arthroplasty (CDA) is an evolving surgical approach for addressing degenerative disc diseases in the cervical spine and has gained attention as an alternative to anterior cervical discectomy and fusion (ACDF). This procedure involves replacing degenerative discs with artificial implants, aiming to preserve motion and potentially mitigate issues commonly associated with fusion, such as adjacent segment degeneration [1, 2]. Several clinical trials have shown promising outcomes in the short term, emphasizing benefits like preserved motion and reduced rates of adjacent segment pathology [3]. However, a comprehensive understanding of the long-term efficacy, safety, and comparative outcomes of cervical disc arthroplasty remains a focus of ongoing research [4, 5]. The need for further investigation into the long-term clinical outcomes, complication rates, and the effects of adjacent-level post-cervical disc arthroplasty is evident [6, 7]. Studies have explored the long-term results of cervical arthroplasty and provided valuable insights into the outcomes and complications; these research efforts aim to provide evidence-based guidelines for clinical decision-making and to enhance patient outcomes by filling the knowledge gaps in the field of cervical disc arthroplasty [8–10]. 

Considering all possible complications of cervical disc diseases, applications of evidence-based medical research become essential when choosing the best treatment method. Statistical approaches that utilize the *P* value reveal the significance of potential divergences between clinical interventions [11]. In the case of CDA, evidence-based medical practice can apply the foundational implications of said statistical results to determine treatment outcomes for damaged cervical discs. Various clinical literature has used the *P* value as a conventional evaluation method for interpreting the marginal significance of statistical results. In recent research findings, novel methodologies have been adopted with the utilization of the fragility index (FI) and fragility quotient (FQ) to assess the robustness of clinical trial results. When evaluating the fragility of randomized controlled trials (RCTs), the FI is to reveal the necessary number required to reverse a trial from high degrees of significance to low or none [12]. In addition, as a measurement of fragility, the FQ further assesses the FI concerning the sample size by dividing the FI by sample size [13]. Combined, the FI and FQ complement the *P* value and promote confidence that low degrees of fragility (higher fragility index) indicate more robust clinical trial data.

Evidence-based medical research will enable physicians to practice evidence-based patient care when treating cervical disc diseases. Despite the debate between conservative and nonconservative management of cervical disc diseases, available literary evidence continues to explore the overall patient outcomes of CDA [14–16]. With these considerations, it becomes even more essential to analyze the robustness of the results in the literature. This study evaluates the degree of statistical fragility in the cervical disc arthroplasty (CDA) literature. We hypothesize that the dichotomous outcomes within the CDA literature are statistically fragile and will be vulnerable to a small number of outcome event reversals.

## Methods and materials

A systematic review was conducted to compare statistical robustness for cervical disc injuries. Since this was a systematic review, no ethical consent or IRB consultation was needed to continue this study. The Preferred Reporting Items for Systematic Reviews and Meta-Analyses (PRISMA) guidelines were used for article identification and consequent selection. The PubMed database was queried from 2000 to 2023, for all RCTs relating to cervical disc arthroplasty. The language was restricted to English. Search criteria involved articles containing: “cervical disc arthroplasty” OR “cervical disc replacement”. Inclusion criteria consisted of RCTs describing dichotomous outcomes with associated *P* values. Studies evaluating non-RCT, non-dichotomous outcomes, systematic reviews, meta-analyses, animal studies, cadaveric studies, biomechanical studies, case reports, greater than two intervention group studies, and cervical disc articles before 2000 were excluded from this review. For the included studies, the following were extracted: journal name, publication year, authors, PubMed Identifier, loss to follow-up (LTF), study design, and *P* values. The journals that met the search criteria included: *Clinical Orthopaedics and Related Research, Clinical Spine Surgery, International Orthopaedics, The Journal of Bone and Joint Surgery, Journal of Neurosurgery: Spine (Phila Pa 1976), Journal of the Pakistan Medical Association, Journal of Spinal Disorders and Techniques, Neurosurgical Focus, Orthopedics*, and *The Spine Journal*.

Study outcomes with levels of statistical significance displayed a *P* value of less than 0.05. Conversely, statistically nonsignificant data had a set *P* value greater than or equal to 0.05. A cross-tabulation was created to compare and categorize the FI of each dichotomous outcome event. The number of outcome events was modified to reverse significance. The FI is defined as the designated number that was required to reverse the significance of patient outcomes (Table 1). The FI was calculated and recorded for each outcome, event, and nonevent. The median FI that incorporated all outcomes was marked as the overall FI for this present study. The FQ was calculated by dividing the FI by the sample size. Characteristics of RCTs were split into subgroups for comparison. FI and FQ subgroups consisted of primary versus secondary outcomes, initial significance (*P* < 0.05 vs. *P* ≥ 0.05), complications, comparing outcomes FI to LTF (FI < LTF vs. FI > LTF), and year (Table 2). Overall FI and FQ were measured by adding all outcome events. FI outcomes were calculated using the two-tailed Fisher exact test. Characteristics organized within each subgroup were calculated via interquartile ranges (IQRs) for FI and FQ. The IQR measures the difference between the upper quartile (Q3, 75th percentile) and lower quartile (Q1, 75th percentile). A risk-of-bias assessment was also performed (Table 3).

**Table 1:** Demonstration of reversal significance with a fragility of 1

	Outcome A	Outcome B	*P* Value
Scenario 1			
Treatment A	13	27	
Treatment B	5	35	**0.059**
Scenario 2			
Treatment A	**14**	**26**	
Treatment B	5	35	**0.034**

**Table 2:** Overall fragility data and analysis of subgroups^a^

Characteristic	Events	Fragility Index (IQR)	Fragility Quotient (IQR)
**All trials**	693	5 (3-7)	0.019 (0.011-0.043)
**Outcome significance** ^b^			
* P* < 0.05	130	5 (2-11)	0.017 (0.006-0.044)
*P >* 0.05	563	5 (4-7)	0.019 (0.011-0.043)
**Comparing outcome FI to LTF** ^c^			
FI *<* LTF	565	5 (3-7)	0.016 (0.009-0.033)
FI > LTF	128	5 (4-7)	0.055 (0.025-0.092)
**Year of publication**			
2000 – 2007	43	5 (4-5)	0.052 (0.043-0.073)
2008 – 2015	434	5 (3-7)	0.016 (0.009-0.036)
2016 – 2023	216	5 (3-10)	0.021 (0.011-0.044)
**Journals**			
Clinical Orthopaedics and Related Research	19	5 (4-7)	0.060 (0.048-0.084)
Clinical Spine Surgery	26	4.5 (4-8)	0.059 (0.040-0.133)
International Orthopaedics	20	4 (4-6)	0.054 (0.045-0.062)
Journal of Bone and Joint Surgery	62	5 (3-6)	0.017 (0.011-0.030)
Journal of Neurosurgery: Spine	156	6 (3-10)	0.025 (0.011-0.045)
Spine (Phila Pa 1976)	246	5 (3-7)	0.014 (0.009-0.024)
Journal of the Pakistan Medical Association	6	4.5 (4-7)	0.107 (0.095-0.167)
Journal of Spinal Disorders and Techniques	42	5 (5-9)	0.043 (0.011-0.067)
Neurosurgical Focus	18	4 (4-5)	0.073 (0.073-0.091)
Orthopedics	5	4 (4-5)	0.167 (0.167-0.208)
The Spine Journal	93	5 (3-8.5)	0.014 (0.008-0.028)

^a^FI, fragility index; IQR, interquartile range; LTF, lost to follow-up

^b^*P<* 0.05 represents the significant outcome subgroup and *P* > 0.05 represents the insignificant outcome subgroup

^c^FI *<* LTF represents the outcome subgroup where the FI was less than the number of patients LTF. FI > LTTF represents the outcome subgroup where the FI was greater than the number of patients LTF

**Fig. 1:**
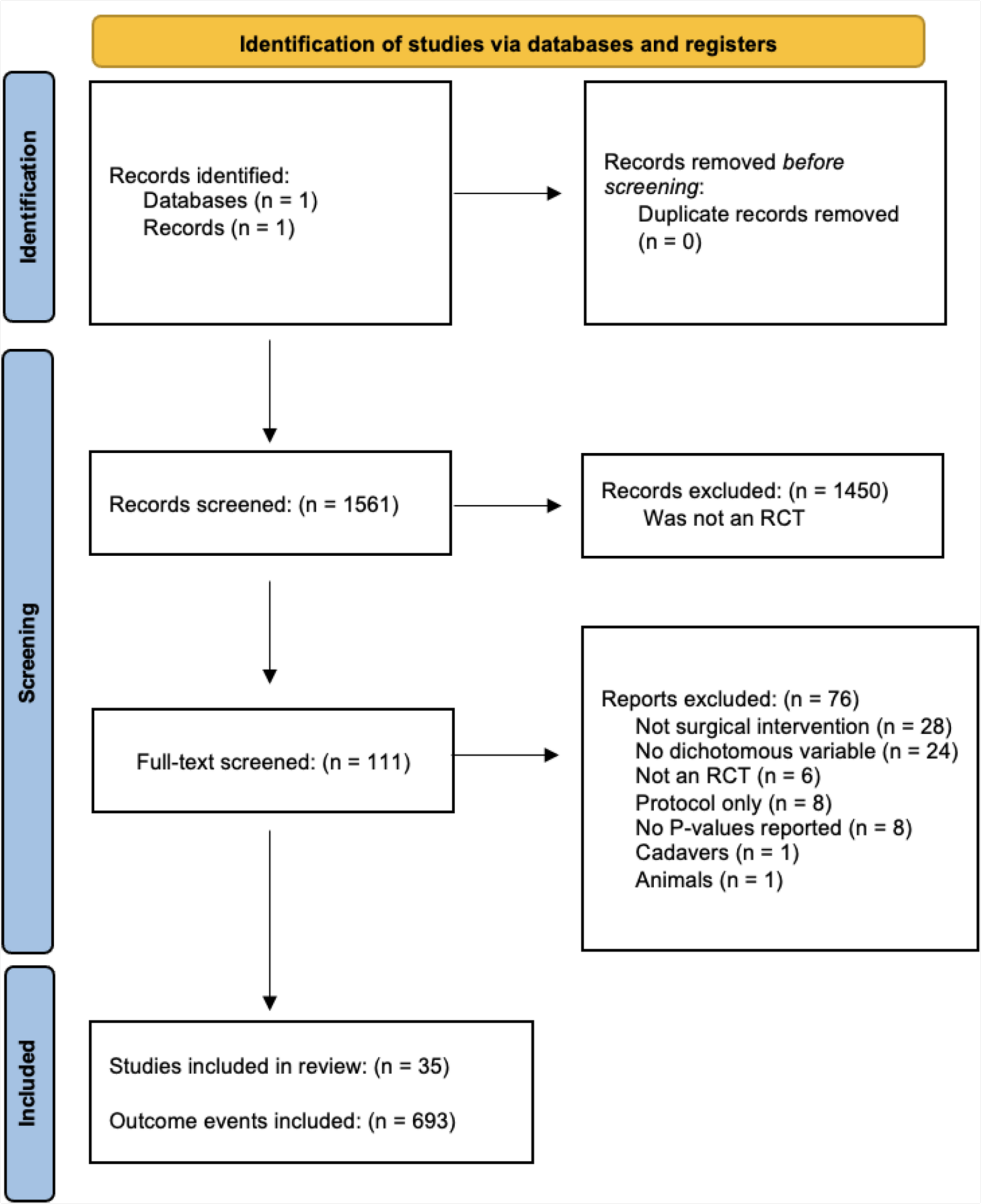
PRISMA flow diagram of included studies

**Table 3:** Risk of bias assessment

	Domain 1: Risk of Bias Arising from Randomization Process	Domain 2: Risk of bias due to deviations from the intended interventions	Domain 3: Risk of Bias Due to Missing Outcome Data	Domain 4: Risk of bias in measurement of the outcome	Domain 5: Risk of bias in selection of the reported result	Overall Risk of Bias
Loidolt et al. [29]	Low Risk	Low Risk	Low Risk	Low Risk	Low Risk	Low Risk
Lombardi et al. [30]	Low Risk	Low Risk	Unclear	Low Risk	Low Risk	Low Risk
MacDowall et al. [31]	Low Risk	Low Risk	Low Risk	Low Risk	Low Risk	Low Risk
Lavelle et al. [32]	Low Risk	Low Risk	Low Risk	Low Risk	Low Risk	Low Risk
Coric et al. [33]	Low Risk	Low Risk	Low Risk	Low Risk	Unclear	Low Risk
Rožanković et al. [34]	Low Risk	Low Risk	Low Risk	Low Risk	Low Risk	Low Risk
Gornet et al. [34]	Low Risk	Low Risk	Low Risk	Low Risk	Low Risk	Low Risk
Lanman et al. [35]	Low Risk	Low Risk	Low Risk	Low Risk	Low Risk	Low Risk
Pandey et al. [36]	Low Risk	Low Risk	Low Risk	Low Risk	Low Risk	Low Risk
Qizhi et al. [37]	Low Risk	Low Risk	Low Risk	Low Risk	Unclear	Low Risk
Radcliff et al. [38]	Low Risk	Low Risk	Unclear	Low Risk	Unclear	Low Risk
Janssen et al. [39]	Low Risk	Low Risk	Unclear	Low Risk	Unclear	Low Risk
Davis RJ et al. [40]	Low Risk	Low Risk	Unclear	Low Risk	Low Risk	Low Risk
Graham et al. [41]	Low Risk	Low Risk	Unclear	Low Risk	Low Risk	Low Risk
Phillips et al. [42]	Low Risk	Low Risk	Low Risk	Low Risk	Low Risk	Low Risk
Skeppholm et al. [43]	Low Risk	Low Risk	Low Risk	Low Risk	High Risk	Low Risk
Zhang et al. [44]	Low Risk	Low Risk	Low Risk	Low Risk	Low Risk	Low Risk
Karabag et al. [45]	Low Risk	Low Risk	Low Risk	Low Risk	Low Risk	Low Risk
Davis et al. [46]	Low Risk	Low Risk	Low Risk	Low Risk	Low Risk	Low Risk
Kang et al. [47]	Low Risk	Low Risk	Low Risk	Low Risk	Low Risk	Low Risk
Phillips et al. [2]	Low Risk	Low Risk	Low Risk	Low Risk	Low Risk	Low Risk
Vaccaro et al. [48]	Low Risk	Low Risk	Low Risk	Low Risk	Low Risk	Low Risk
Zigler et al. [49]	Low Risk	Low Risk	Low Risk	Low Risk	Unclear	Low Risk
Zhang et al. [50]	Low Risk	Low Risk	Low Risk	Low Risk	Low Risk	Low Risk
Cheng et al. [51]	Low Risk	Low Risk	Low Risk	Low Risk	Low Risk	Low Risk
Sasso et al. [7]	Low Risk	Low Risk	Low Risk	Low Risk	Low Risk	Low Risk
Garrido et al. [52]	Low Risk	Low Risk	Low Risk	Low Risk	Low Risk	Low Risk
Cheng et al. [53]	Low Risk	Low Risk	Low Risk	Low Risk	Low Risk	Low Risk
Heller et al. [3]	Low Risk	Low Risk	Low Risk	Low Risk	Low Risk	Low Risk
Phillips et al. [54]	Low Risk	Low Risk	Low Risk	Low Risk	Low Risk	Low Risk
Sasso et al. [55]	Low Risk	Low Risk	Low Risk	Low Risk	Low Risk	Low Risk
Anderson et al. [56]	Low Risk	Low Risk	Low Risk	Low Risk	Low Risk	Low Risk
Sasso et al. [57]	Low Risk	Low Risk	Low Risk	Low Risk	High Risk	Low Risk
Sasso et al. [58]	Low Risk	Low Risk	Low Risk	Low Risk	Low Risk	Low Risk
Porchet et al. [59]	Low Risk	Low Risk	Low Risk	Low Risk	Low Risk	Low Risk

## Results

Of the 1561 studies screened, 111 met the search criteria with 35 RCTs included in the analysis (Fig. 1). A total of 693 events with 130 significant (*P* < 0.05) outcomes and 563 with nonsignificant (*P* > 0.05) outcomes were identified. The overall FI, incorporating all 693 outcome events from the 35 RCTs was 5 (IQR 3–7). The overall FQ was 0.019 (IQR 0.011–0.043), indicating that the reversal of 13 of 100 outcomes may change the study significance of the included RCTs. Of the 9 included RCTs, 3 studies reported loss to follow-up (LTF) data greater than the overall FI of 5. Therefore, 33.3% of studies reported an LTF value that was greater than the overall FI. For the 130 outcomes that were reported as significant, the median FI of events required to change significance was 5 (IQR 2–11) (Table 2). The FQ for significant outcomes was 0.017 (IQR 0.006–0.044). For the 563 outcomes that were reported as nonsignificant, the number of events required to change significance was 5 (IQR, 4–7). The FQ for nonsignificant outcomes was 0.019 (IQR 0.011–0.043). For the outcomes where FI *≤* LTF (*n* = 565), the median FI was found to be 5 (IQR 3–7). For the outcomes where FI > LTF (*n* = 128), the median FI was found to be 5 (IQR 4–7). The associated median FQs were 0.016 (IQR 0.009–0.033) and 0.055 (IQR 0.025–0.092), respectively. Fragility subanalysis per year of publication identified an FI of 5 (IQR 4–5) from 2000 to 2007, an FI of 5 (IQR 3–7) from 2008 to 2015, and an FI of 5 (IQR 3–10) from 2016 to 2023, thus demonstrating consistent statistical fragility over the 23 years.

## Discussion

Our fragility analysis exploring the cervical disc arthroplasty revealed an overall median FI of 5 (IQR 3–7) and a median FQ of 0.019 (IQR 0.011–0.043). A fragility index of 5 indicates that 5 events were needed to alter the statistical significance from significant to nonsignificant or vice versa. In correlation with sample size, the FQ of 0.019 demonstrates that 2 out of 100 patients is necessary for the median number to reverse significance across the total outcomes. In the RCTs included in this paper, 65.7% (23) of studies did not report or reported an LTF greater or equal to an overall FI of 5. Through this finding, LTF maintenance is an important tool that can help increase the overall robustness of a given study. The low median FI and FQ depict the statistical fragility within the CDA literature as only a small number of events is necessary to alter statistical significance. The application of a fragility analysis gives us an objective measure to compare clinical robustness where a higher FI would increase the confidence in the effectiveness of the data depicted in that study. This metric simply allows us to assess the quality of the studies that guide management algorithms in evidence-based medicine and accurately interpret clinical data that guide clinical care.

In this systematic review, the overall FI of 5 reported was similar to many of the previous spine and orthopedic literature related to statistical fragility. Evaniew et al. were the first to apply a fragility index to the spine literature and reported an overall median FI of 2 [17]. In a recent study, Muthu et al. reexamined the spine literature and reported an overall median FI of 2 [18]. However, these studies focus on the overall spine literature and demonstrate significant fragility while our study highlights CDA literature to be more robust compared to the previous analyses. Gupta et al. examined the use of ketamine infusion during scoliosis surgery and reported a FI of 2. Cordero et al. calculated the statistical fragility of tourniquet use in total knee arthroplasty (TKA) and obtained an FI of 4 [19]. The anterior cruciate ligament reconstruction (ACLR) literature comparing bone-patellar tendon-bone grafts and hamstring tendon autografts revealed a FI of 5 [20]. When looking at autograft vs. allograft comparison in ACLR, the authors found a FI of 6 [21]. A recent fragility analysis looking at calcaneus fractures demonstrated an FI of 6 as well [22]. In an examination of the distal biceps repair literature, the authors demonstrated an FI of 6.5 [23]. Megafu and Megafu in examining the distal radius fracture literature reported an FI of 9, the highest FI within all orthopedic and spine literature to date [24]. Other trauma literature looking at fibula fractures, distal femur fractures, and orbital fractures reported a similar FI of 5 across all studies [25–27]. By the spine and orthopedic literature, the FI consistently demonstrates statistical fragility, thus confirming our hypothesis and continual evidence of fragile results within the spine and orthopedic literature.

This review paper analyzing the spine literature regarding cervical disc arthroplasty has many strengths and limitations. An inherent strength in this review is the unbiased, comprehensive nature of having a systematic review of RCTs with the utilization of the PRISMA methodology. Also, the sole use of RCTs is a notable strength as management protocols and evidence-based medicine guidelines derive their decision-making abilities based on these studies. However, it is important to acknowledge this study’s limitations. The first potential weakness is that fragility analysis is solely applicable to binary data with dichotomous endpoints. This limits FI utility for non-dichotomous studies. Studies with ordinal and continuous outcomes were filtered out to prevent overlap. Adding more study designs may have influenced the ability to further investigate CDA results. However, our team was able to comprehensively assess all possible binary outcomes by utilizing a dichotomous approach. Another limitation was that studies needed to be RCTs to be eligible for inclusion in this present review. As a result cohort studies, case-control studies, cross-sectional studies, prospective studies, and retrospective studies were excluded from analysis. Despite reduced incorporation of subject outcome events, our systematic review with RCTs provided a suitable, reliable way to assess effective CDA treatment options.

With the ever-expanding advancements in medicine, our systematic review aimed to quantify robustness in CDA literature by exploring the FI and FQ values to confirm the clinical significance of CDA. Our findings show that the majority of RCTs in cervical disc arthroscopic surgery have consistently been less robust for the last 23 years as speculated by our team. Possible implications for this loss of statistical significance may be due to solitary reliance on *P* value analysis, which inadvertently overlooks LTFs, impacts of varying sample size, and event outcomes [28]. Integrating fragility analysis in conjunction with *P* value analysis can be helpful when trying to carefully measure the robustness of medical procedures. Due to the recent development of fragility analysis, it would be in the clinician’s best interest to further support clinical data with additional statistical tools, such as the minimal clinically important difference (MCID), substantial clinical benefit (SCB), and patient-acceptable symptomatic state (PASS). These research tools can be used to leverage FI and FQ data to strengthen understanding of relationships and patterns for significant outcomes in respective trials.

## Conclusion

The literature regarding CDA RCTs lacks statistical robustness and may misrepresent the conclusions with the sole use of the *P* value. By implementing of the FI and FQ along with the *P* value, we believe the interpretation and contextualization of the clinical data surrounding CDA will be better understood.
